# Supplementary material for: Brd4‐Brd2 isoform switching coordinates pluripotent exit and Smad2‐dependent lineage specification
Source: EMBO Rep. 2017 Jun 6;18(7):1108–22. doi: 10.15252/embr.201643534 (PMC5494510; doi:10.15252/embr.201643534)
Supplement: Supplementary file 1 — Appendix [file EMBR-18-1108-s001.pdf]

Appendix

Appendix Table S1: Summary of Small Molecule Screen **p1**
Appendix Table S2: List of primary antibodies **p2**
Appendix Table S3: Primers used for qRT-PCR **p3**
Appendix Table S4: Primers used for ChIP analyses **p4**

Appendix Table S1

Compound number	Compound	Screening Concentration (uM)	Primary target	RNA concentration (ng/ml)	Brachyury fold change/DMSO control
1	A-83-01	1	ALK5	475.07	0.001
2	MSC 2032964A	1	ASK1	584.65	1.05
3	KU55933	1	ATM	702.8	0.79
4	ETP 46464	1	ATRi	477.22	1.86
5	SU-6668	1	Aurora	710.19	0.35
6	VX-680	1	Aurora	744.48	0.59
7	PLX 4720 (Raf Kinase Inhibitor V)	1	B-Raf	622.79	0.97
8	Gleevec (Imatinib)	1	BCR-ABL	666.43	0.62
9	Dasatinib	1	BCR-ABL, Src	0	N/A
10	LDN-193189	1	BMP	770.7	1.27
11	LFM-A13	1	BTK	634.2	0.93
12	PCI-32765 (Ibrutinib)	1	Btk	287.46	3.1
13	PD 0332991	1	CDK4, CDK6	679.15	0.74
14	CKI-7	1	CK1	674.35	0.38
15	D 4476	1	CK1	818.17	0.38
16	IC261	1	CK1	0	N/A
17	GSK626616	1	DYRK	425.19	0.5
18	AG1478 (Tyrphostin)	1	EGFR	528.48	0.54
19	AG490	1	EGFR, JAK	565.05	0.64
20	(Z)-4-Hydroxy tamoxifen	1	Estrogen receptor	565.95	0.94
21	Zearalenone	1	Estrogen receptor (ER)	303.76	1.09
22	CHIR 98014 (CT 98014)	1	GSK3	546.62	0.43
23	CHIR 99021 (CT 99021)	1	GSK3	623.9	0.94
24	BI605906 (BIX02514)	1	IKKβ	741.43	0.53
25	IRAK-4 kinase inhibitor a	1	IRAK4	457.2	0.5
26	CP-690550 (Tasocitinib)	1	JAK	477	0.2
27	Ruxolitinib (INCB18424)	1	JAK	612.24	0.39
28	JNKIN7	1	JNK	523.56	0.89
29	JNKIN8	1	JNK	635.92	0.52
30	GSK2578215A	1	LRRK2	684.4	0.5
31	HG-10-102-01	1	LRRK2	698.21	0.52
32	MAPKAP-K2 A	1	MAPKAP-K2	535.52	0.69
33	MAPKAP-K2 B	1	MAPKAP-K2	572.67	0.45
34	PF3644022	1	MAPKAPK2	578.7	0.49
35	MRT199665	1	MARK	0	N/A
36	PD 0325901	1	MEK1	596.1	0.79
37	ML-9, Hydrochloride	1	MLCK	596.56	4.37
38	CGP-57380	1	MNK	581.96	2.16
39	CGP-57380 analog (SHN-093)	1	MNK	710.36	1.93
40	AZD8055	1	mTOR C1, mTOR C2	0	N/A
41	Amgen-NIK-28	1	NIK	0	N/A
42	BIRB-0796 (Doramapimod)	1	p38 MAPK	601.78	0.51
43	GSK2334470	1	PDK1	467.49	1.37
44	GSK2606414	1	PERK	655.51	0.74
45	GDC-0941	1	PI3K	0	N/A
46	H-89, Dihydrochloride	1	PKA	715.17	0.75
47	MK-2206	1	PKB	381.736	1.72
48	AX 20017	1	PknG	737.16	0.94
49	BI 2536	1	PLK1	0	N/A
50	GSK461364	1	PLK1	0	N/A
51	Necrostatin-1 (Nec-1)	1	RIP	501.57	1.56
52	Nectrostatin-1 (Nec-1 inactive analog)	1	RIP	618.83	0.61
53	GSK429286	1	ROCK	671.51	0.87
54	BI-D-1870	1	RSK	584.99	0.05
55	SL0101-1	1	RSK	667.02	0.44
56	PF4708671	1	S6K1	766.1	0.42
57	HG-9-91-01	1	SIK	0	N/A
58	BML-258 (SK1-I)	1	SphK1	542.4	0.73
59	Sphingosine Kinase Inhibitor (SKI II)	1	SphK1	455.01	1.14
60	(5Z)-7-Oxozeaenol	1	TAK1	413.98	1.57
61	NG-25	1	TAK1	663.56	0.57
62	LY364947	1	TGF-β type-I	668.45	0.09
63	VPS34-IN1	1	Vps34	646.01	0.67
64	STOCK1S-50699	1	WNK	298.66	0.14
65	Cot-Tpl2 Inhibitor Compound 3 (Wyeth)	1	Cot-Tpl2	530.95	0.84
66	Cot-Tpl2 Inhibitor Compound 31 (Wyeth)	1	Cot-Tpl2	476.65	0.76
67	401 KuDOS (Compound 401)	1	DNA-PK	639.75	0.54
68	Celastrol	1	hsp90, ampk	594.7	1
69	MRT67307	1	IKKε, TBK1	666	0.94
70	HTH-01-015	1	NUAK1	664.5	0.57
71	IPA-3	1	PAK1	600.84	0.69
72	Rigel TRAF6 Inhibitor	1	TRAF6	636.73	0.36
73	Princeton's TrkA inhibitor compound 20h	1	Trk A	762.88	0.27
74	AstraZeneca's Trk A	1	Trk A, Trk B	0	N/A
75	ML 307	1	UBC 13	669.76	0.32
76	LDN57444	1	UCH-L1	481.59	0.31
77	UCH-L1 inhibitor (Compound 1)	1	UCH-L1	796.64	0.98
78	UCH-L1 inhibitor (Compound 3)	1	UCH-L1	720.5	0.49
79	UCH-L3 inhibitor (Compound 10)	1	UCH-L3	640.11	0.53
80	IU1	1	USP14	583.04	1.09
81	WP1130 (Degrasyn)	1	USP7	560.44	0.52
82	USP8 Inhibitor (Compound 15)	1	USP8	632.22	1.24
83	USP8 Inhibitor (Compound 16)	1	USP8	668.56	1.59
84	Resveratrol	1	cyclo-oxygenase 1	583.24	2.39
85	MLN4924	1	NEDD8-activating enzyme	691.12	0.05
86	BAY-11-7082	1	NFKBIA	637.13	0.38
87	KF1B	1	Nod1	649.39	0.51
88	M-Tri-DAP	1	Nod1	612.05	0.59
89	ML240	1	p97 ATP	0	N/A
90	ML241	1	p97 ATP	393.02	0.25
91	Novartis 12a (PKD1)	1	PKD1	668.78	0.97
92	MLN9708	1	Proteasome	0	N/A
93	SRT 1720	1	SIRT1	0	N/A
94	Smac mimic (compound 3)	1	Smac mimic (active)	745.07	0.6
95	Smac mimic inactive (compound 4)	1	Smac mimic (inactive)	623.61	0.45
96	NU 9056	1	Tip60, KAT5	616.34	0.54
97	VCP-c-116	1	VCP	0	N/A
98	Febuxostat (TMX-67, Adenuric, Uloric)	1	Xanthine oxidase (XO)	666.17	0.36
99	DP00966	1	FEN1	726.99	0.48
100	Blebbistatin	1	myosin II	632.4	0.5
101	Ischemin	1	p53 CBP	0	N/A
102	DBeQ (KSC-16-67)	1	p97 inhibitor	616.13	0.7
103	CCT 007093	1	PPM1D	702.73	0.66
104	Harmaline	1	serotonin, monoamine oxidase	711.57	0.76
105	JQ1	0.1	BET bromodomains	465.58	0.0004

Appendix Table S2: List of primary antibodies

**Western Blotting**	
ppERK	CST#9101
Erk1/2	Santa Cruz Biotechnology# sc-93
Phospho-β-catenin	CST#9561
β-catenin	Santa Cruz Biotechnology# sc-7963
Phospho-Smad2	CST#3108
Smad2	CST#5339
Phospho-Smad1/5	CST#9516
Smad1	CST#9743
Brachyury	AbGent#AM1993
Nanog	Reprocell#RCAB001P
Brd2	A302-583A
Brd3	Abcam#ab50818 [2088C3a]
Brd4 (WB)	Abcam#ab128874
Lamin B1	Proteintech 66095
**Immunostaining**	
FoxA2 (HNF-3 beta)	Santa Cruz Biotechnology# sc-271103
**ChIP**	
Acetyl-Histone H4	Millipore 06-866
Trimethyl-histone H3	Millipore 07-473
Cdk9	Santa Cruz Biotechnology# sc-484
Brd2	A302-583A
Brd4	Malik et al, 2015

Appendix Table S3: Primers used for qRT-PCR

**Mouse**		
**Gene**	**Forward**	**Reverse**
Klf4	ACACTTGTGACTATGCAGGCTGTG	TCCCAGTCACAGTGGTAAGGTTTC
Fgf5	GCTGTGTCTCAGGGGATTGT	CACTCTCGGCCTGTCTTTTC
Nestin	AACTCTCGCTTGCAGACACCTG	AGGTGCTGGTCCTCTGGTATCC
Sox1	TTCCCCAGGACTCCGAGGCG	GCTGTGTGCCTCCTCTGCGG
Brachyury	TCCCGAGACCCAGTTCATAG	TTCTTTGGCATCAAGGAAGG
Mixl	GCTGCTACCCGAGTCCAGGAT	GCCTTGAGGATAAGGGCTGAAA
Goosecoid	GGAGACGAAGTACCCAGACG	AAACCAGACCTCCACCTTCTC
Nodal	GGCGTACATGTTGAGCCTCT	CGTGAAAGTCCAGTTCTGTCC
Inha	AGGAAGATGTCTCCCAGGCT	GGATGGCCGGAATACATAAG
Inhba	GATCATCACCTTTGCCGAGT	CACTTCTGCACGCTCCACTA
Inhbb	CTAGAGTGTGATGGGCGGAC	GCGCAATGATCCAGTCGTTC
TGFb1	GCCCTGGATACCAACTATTGC	AAGTTGGCATGGTAGCCCTT
Alk5	TCTGCATTGCACTTATGCTGA	AAAGGGCGATCTAGTGATGGA
Alk7	TCTGGTCTGCCTCTCTTGGT	AGCCACATCTTCTCCACACC
Acvr2a	ACACAGCCCACTTCAAATCC	AGGAGGGTAGGCCATCTTGT
Acvr2b	AGGGCCACAAGCCTTCTATT	CCAACCTGTCCATGGGTATC
TGFβR2	AACATGGAAGAGTGCAACGAT	CGTCACTTGGATAATGACCAACA
Smad2	ATGTCGTCCATCTTGCCATTC	AACCGTCCTGTTTTCTTTAGCTT
Cripto	TTCTCAGCCCTGTACTGCCT	CTTGGGATGCTGCATTCTCT
Lefty1	CTGCTACAACACAGCCATGC	GGAGGTCTCTGACACCAGGA
Lefty2	CATGAAGTCCCTGTGGCTTT	ATGGCCATCTCCTCCACAT
GAPDH	CTCGTCCCGTAGACAAAA	TGAATTTGCCGTGAGTGG
Nascent Nodal	GGCGTACATGTTGAGCCTCT	TCCCAGTCTCTGGACGATTC
**Human**		
**Gene**	**Forward**	**Reverse**
Brachyury	TATGAGCCTCGAATCCACATAGT	CCTCGTTCTGATAAGCAGTCAC
Sox17	TTCGTGTGCAAGCCTGAGATG	GTCGGACACCACCGAGGAA
FoxA2	GGAGCAGCTACTATGCAGAGC	CGTGTTCATGCCGTTCATCC
Nodal	GGGACTCGGTGGGGCTGGTAACGT	GGGCAAGAGGCACCGTCGACATCA

Appendix Table S4: Primers used for ChIP analyses

**Mouse**		
**Gene**	**Forward**	**Reverse**
Nodal -3.3 (AIE)	GGGGAAGCACCATGAGTTGA	CCCATGTCCAAGACACCTGA
Nodal -2.0 (HBE)	TGGGGACACATCCTACTAGGT	TCAGAAGTGGAATTTGGAGAGAG
Nodal -0.5	GGATGAACTGGAGAGGACCG	CTACCTCGGGAAGTCTCCCT
Nodal 0.0 TSS	CCTGTTGGGCTCTACTCCAC	GAGGCTCAACATGTACGCCA
Nodal 1.0 (ASE)	CCCAGCGACCTGAGTGATG	CCCAAGAGGCGAGATGTTGA
Negative region	AACCTCACACACAACAAGCTG	TGTGATAGGGAGAATGCTTGC
