# Supplementary material for: Brd4‐Brd2 isoform switching coordinates pluripotent exit and Smad2‐dependent lineage specification
Source: EMBO Rep. 2017 Jun 6;18(7):1108–22. doi: 10.15252/embr.201643534 (PMC5494510; doi:10.15252/embr.201643534)
Supplement: Supplementary file 2 — Expanded View Figures PDF [file EMBR-18-1108-s002.pdf]

Expanded View Figures

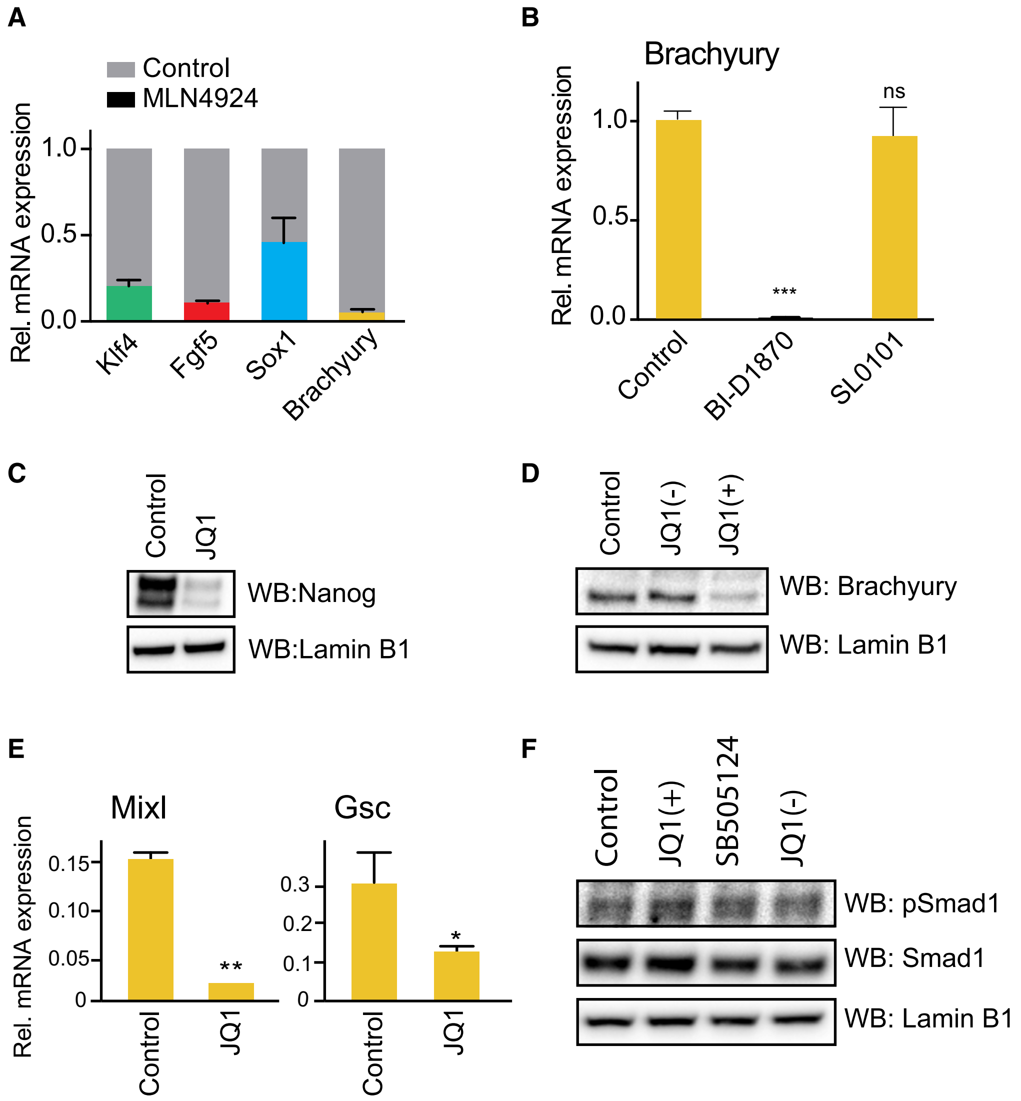

Figure EV1. BET bromodomain activity is required for mesendoderm differentiation.

A mESCs differentiating upon 2i release for 1.5 days were treated with 1 μM MLN4924 for a further 2.5 days. Klf4, Fgf5, Sox1 and Brachyury mRNA levels were determined by qRT–PCR and compared to DMSO control (grey shade). Data are presented as mean ± SD of technical replicates from two experiments.

B mESCs differentiating upon 2i release for 1.5 days were treated with 1 μM BI-D1870 or SL0101 for a further 2.5 days and Brachyury mRNA level determined by qRT–PCR. Data presented as mean ± SD of technical replicates from two experiments; statistical significance was determined using two-tailed unpaired Student's *t*-test (ns = not significant and ***$P < 0.001$).

C 2i mESCs were treated with 100 nM JQ1 or DMSO control for 24 h. Nanog and Lamin B1 protein levels were determined by immunoblotting.

D mESCs differentiating upon 2i release for 1.5 days were treated with 100 nM JQ1(+) or the inactive stereoisomer JQ1(–), for a further 2.5 days. Brachyury and Lamin B1 levels were evaluated by immunoblotting.

E mESCs differentiating upon 2i release for 1.5 days were treated with 100 nM JQ1 for a further 2.5 days, and mRNA expression of Mixl and Goosecoid was determined by qRT–PCR. Data are presented as mean ± SD of technical replicates from two experiments; statistical significance was determined using two-tailed unpaired Student's *t*-test (*$P < 0.05$, **$P < 0.01$).

F mESCs differentiating for 1.5 days were treated with 100 nM JQ1(+), the inactive stereoisomer JQ1(–) or 3 μM SB505124 for a further 2.5 days. Phospho-Smad1, Smad1 and Lamin B1 levels were evaluated by immunoblotting.

Source data are available online for this figure.

 © 2017 The Authors

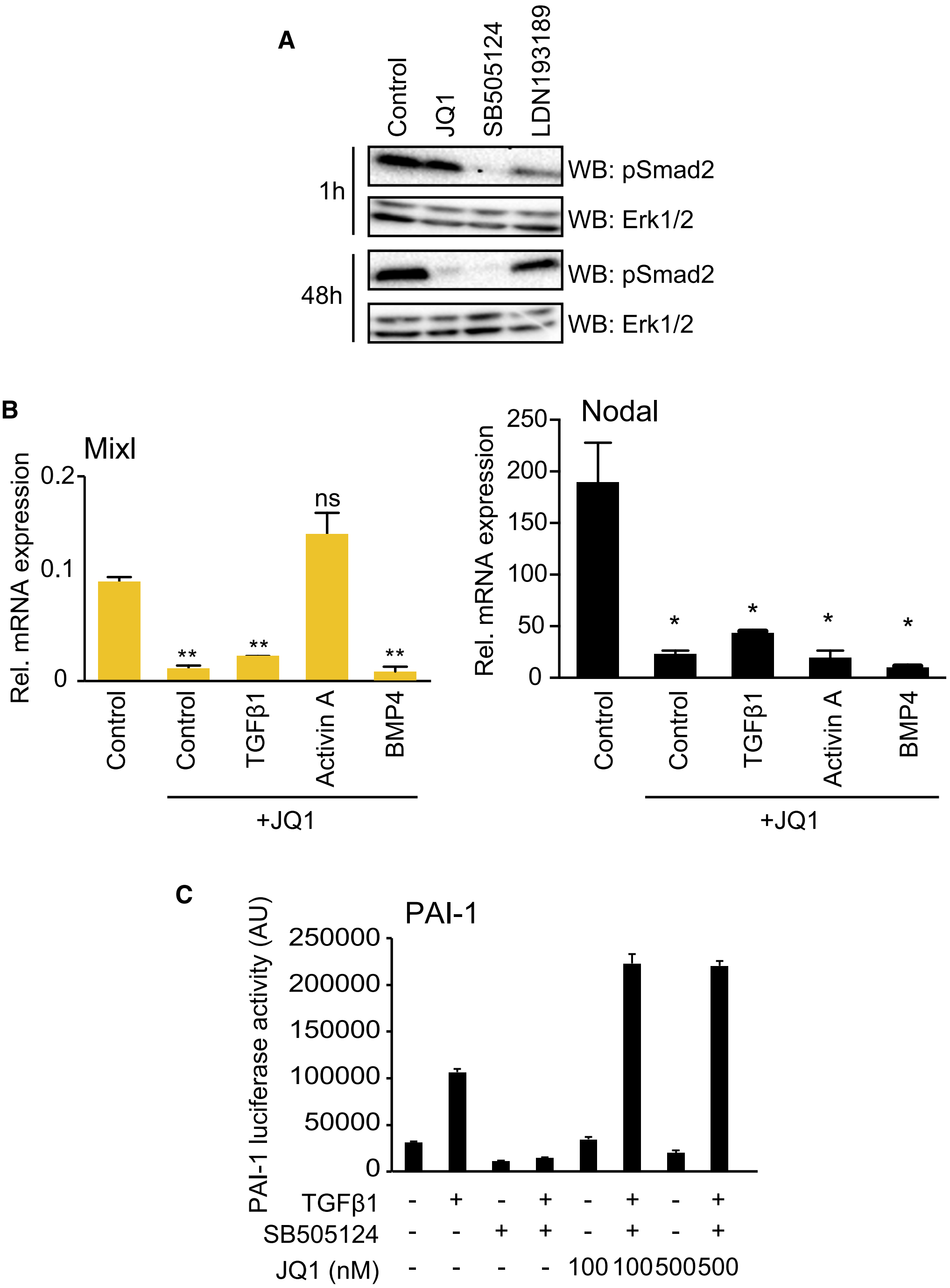

Figure EV2. Specific involvement of BET bromodomain activity in Activin/Nodal signalling.

A mESCs differentiating upon 2i release were treated with 100 nM JQ1, 3 μM SB505124 or 1 μM LDN193189 for 1 h or 48 h. pSmad2 and Erk1/2 levels were determined by immunoblotting.

B mESCs differentiating upon 2i release were treated with 100 nM JQ1 and stimulated with TGFβ1, Activin A or BMP4 for 48 h. Mixl and Nodal mRNA levels were quantified by qRT–PCR. Data are presented as mean ± SD of technical replicates from a representative experiment; statistical significance was determined for each condition relative to control using two-tailed unpaired Student's *t*-test (ns = not significant, $*P < 0.05$ and $**P < 0.01$). Similar results were found in three independent experiments ($n = 3$).

C PAI-1 luciferase U2OS cells were treated with vehicle control, SB505124 or JQ1 for 48 h, stimulated with TGFβ1 and PAI-dependent luminescence determined. Data are presented as mean ± SD of technical triplicates from three experiments ($n = 3$).

Source data are available online for this figure.

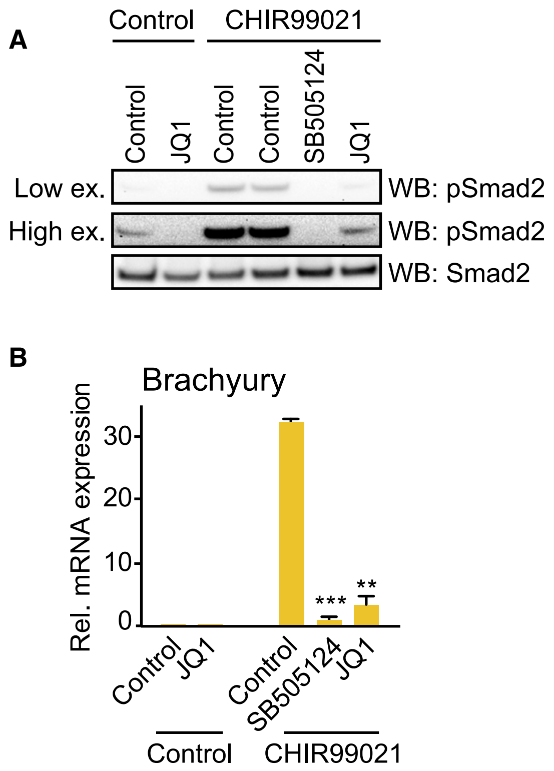

Figure EV3. BET activity is required for Smad2 signalling and Brachyury induction in hiPSCs.

A, B Control hiPSCs or hiPSCs induced to differentiate with CHIR99021 for 24 h were treated with JQ1 or SB505124. (A) Levels of Smad2 phosphorylation and Smad2 were evaluated by immunoblotting. (B) Total RNA was extracted and levels of Brachyury mRNA were quantified by qRT-PCR. Graph shows mean ± SD of technical replicates from a representative experiment; statistical significance was determined using two-tailed unpaired Student's *t*-test (**$P < 0.01$, ***$P < 0.001$). Similar results were found in three independent experiments ($n = 3$).

Source data are available online for this figure.

 © 2017 The Authors

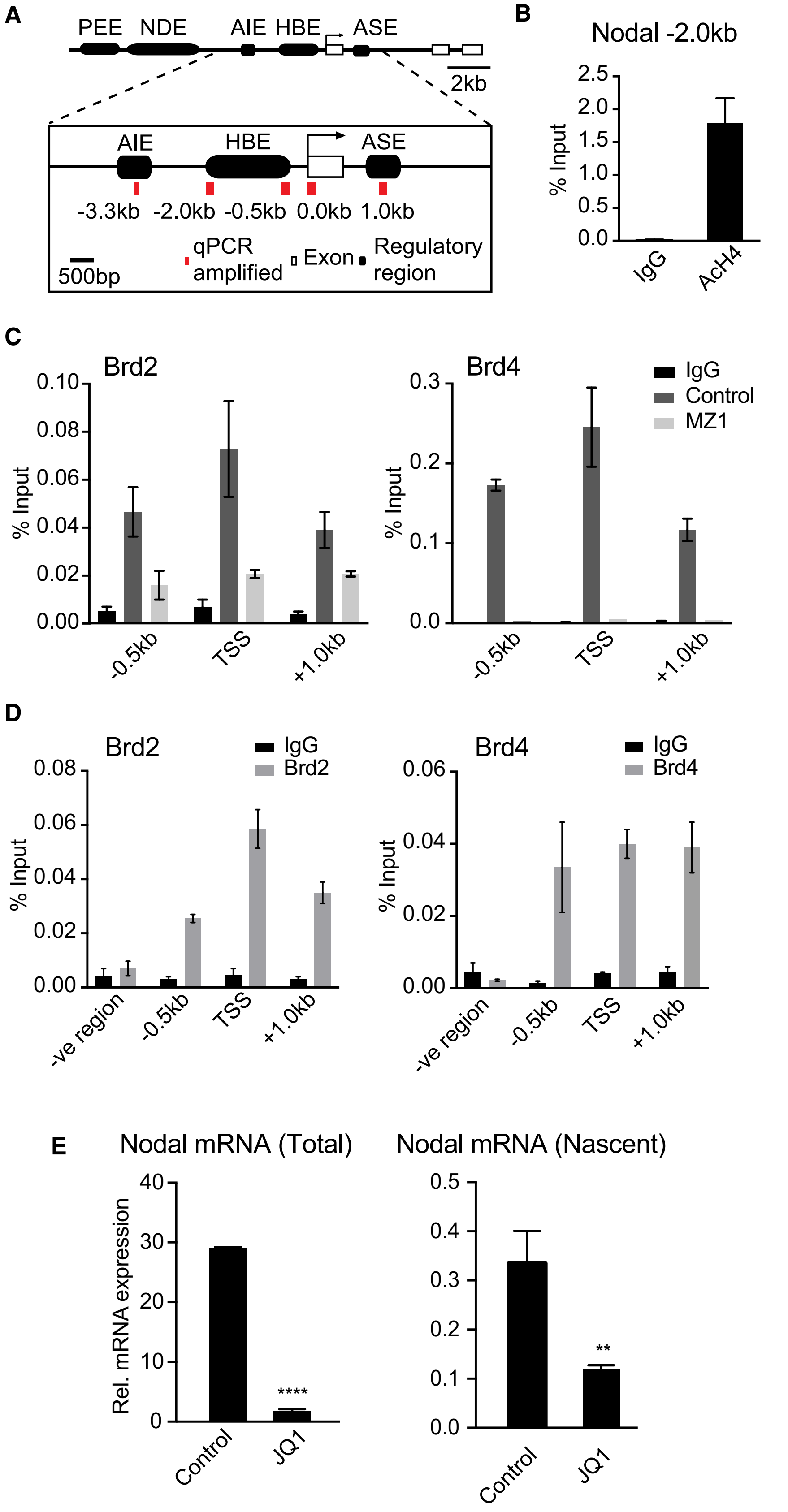

Figure EV4. Direct regulation of Nodal transcription by Brd2-Brd4.

A Nodal regulatory regions, adapted from [32]; PEE = proximal enhancer element, HBE = highly bound element, ASE = asymmetric enhancer, NDE = nodal enhancer, AIE = asymmetric initiator element.

B ChIP was performed on mESCs differentiating upon 2i release using anti-acetyl K5/K8/K12/K16 histone H4 antibody or IgG control. Nodal HBE abundance was quantified by qPCR and the percentage of ChIP DNA compared to input calculated. Data are presented as mean ± SEM of technical replicates from three experiments ($n = 3$).

C mESCs differentiating upon 2i release (Brd2) or 2i mESCs (Brd4) were treated with 1 μM MZ1 for 5 h and ChIP performed using Brd2 and Brd4 antibodies and respective IgG controls. Abundance of NRE sequences was quantified by qPCR and the percentage of ChIP DNA compared to input calculated. Data are presented as mean ± SEM of technical replicates from a representative experiment. Similar results were found in three independent experiments ($n = 3$).

D ChIP was performed on mESCs differentiating upon 2i release using Brd2 and Brd4 antibodies and respective IgG controls. Abundance of NRE and a distal non-binding genomic sequence (–ve region) was quantified by qPCR, and the percentage of DNA enriched compared input was calculated. Data are presented as mean ± SEM of technical replicates from a representative experiment. Similar results were found in three independent experiments ($n = 3$).

E mESCs differentiating for 1.5 days were treated with 100 nM JQ1 for a further 2.5 days, and levels of Nodal nascent mRNA and mature mRNA determined by qRT-PCR. Graph shows mean ± SD of technical replicates from two independent experiments; statistical significance was determined using two-tailed unpaired Student's *t*-test (**$P < 0.01$, ****$P < 0.0001$).

© 2017 The Authors

Figure EV5. Specific requirement for Brd2 in mesendoderm differentiation.

A 2i mESCs were transfected with control, Brd2, Brd3 or Brd4 siRNAs and differentiated in 2i release for 3 days. Sox1 mRNA level was determined by qRT-PCR. Box plots show technical replicates from three independent experiments. Boxes extend from the 25th to the 75th percentile, with the median represented as the line segment inside each box. The whiskers expand from the minimum to the maximum value. Statistical significance was determined for each condition relative to control using two-tailed unpaired Student's *t*-test. (*****P* < 0.0001, ns = not significant).

B 2i mESCs were transfected with control, Brd2, Brd3 or Brd4 siRNAs and differentiated in 2i release for 3 days. Mixl, Lefty1 and Lefty2 mRNA levels were determined by qRT-PCR. Box plots show technical replicates from at least three independent experiments. Boxes extend from the 25th to the 75th percentile, with the median represented as the line segment inside each box. The whiskers expand from the minimum to the maximum value. Statistical significance was determined for each condition relative to control using two-tailed unpaired Student's *t*-test (ns = not significant, **P* < 0.05, ***P* < 0.01, ****P* < 0.001).

C 2i mESCs were transfected with control, Brd2, Brd3 or Brd4 siRNAs and differentiated in 2i release for 3 days. Brd2, Brd3 and Brd4 protein levels were determined by immunoblotting and quantified using ImageLab software. Data are presented as mean ± SD of values obtained in two different experiments ($n = 2$).

D 2i mESCs were transfected with control, Brd2 or Brd4 siRNAs and differentiated in 2i release for 3 days. Nodal and Brachyury mRNA levels were determined by qRT-PCR. Box plots show technical replicates from at least three independent experiments. Boxes extend from the 25th to the 75th percentile, with the median represented as the line segment inside each box. The whiskers expand from the minimum to the maximum value. Statistical significance was determined for each condition relative to control using two-tailed unpaired Student's *t*-test (ns = not significant, **P* < 0.05, ***P* < 0.01, ****P* < 0.001).

 © 2017 The Authors

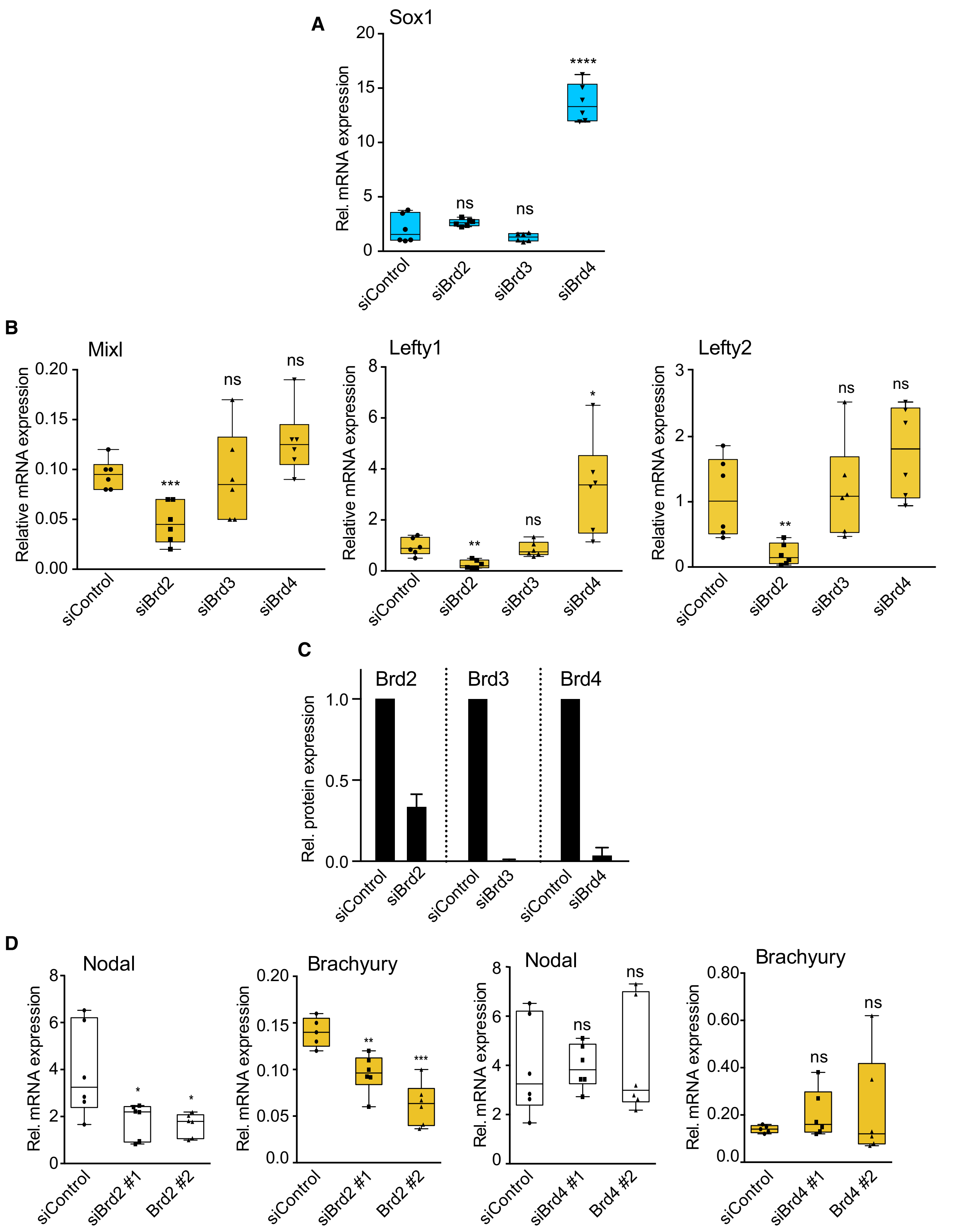

Figure EV5.

© 2017 The Authors
