# Supplementary material for: Brd4‐Brd2 isoform switching coordinates pluripotent exit and Smad2‐dependent lineage specification
Source: EMBO Rep. 2017 Jun 6;18(7):1108–22. doi: 10.15252/embr.201643534 (PMC5494510; doi:10.15252/embr.201643534)

Figure EV1C

Gel: NuPAGE™ 4-12% Bis-Tris Protein Gels, 1.0 mm, 10-well.
Size marker: Precision Plus Biorad #161-0373.
Geldoc XR Plus (Biorad) was used for detection.
ECL (relevant exposures)+ colorimetric overlays are shown unless otherwise stated.

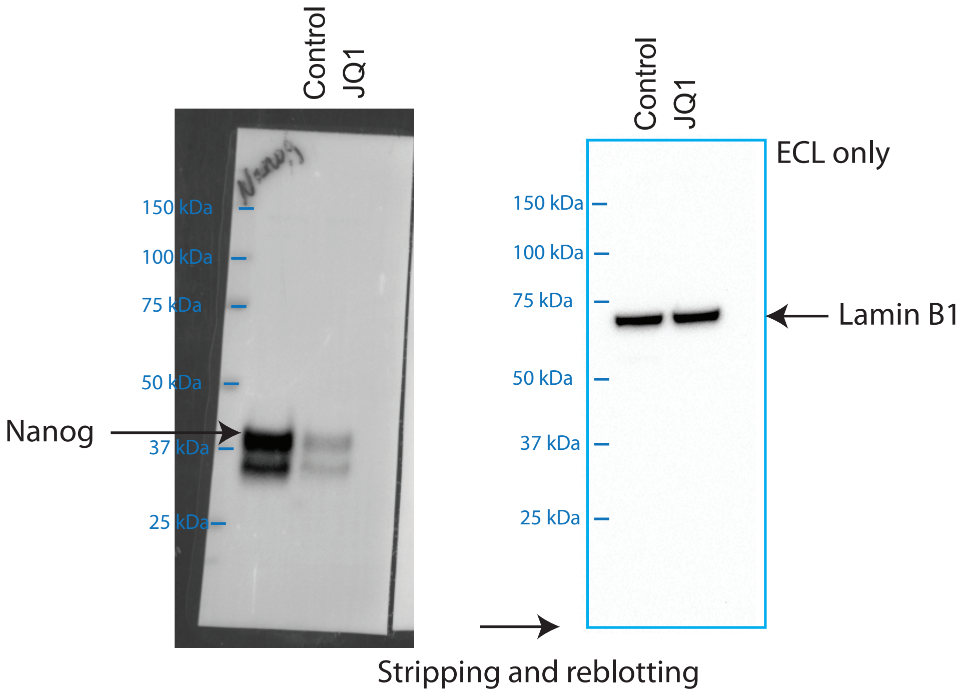

Supplement: Supplementary file 3 — Source Data for Expanded View [file EMBR-18-1108-s008.zip › EV1C_SD.pdf]
