# Supplementary material for: Brd4‐Brd2 isoform switching coordinates pluripotent exit and Smad2‐dependent lineage specification
Source: EMBO Rep. 2017 Jun 6;18(7):1108–22. doi: 10.15252/embr.201643534 (PMC5494510; doi:10.15252/embr.201643534)

Figure EV2A

Gel: 10% Acrylamide (20 wells).
Size marker: Precision Plus Biorad #161-0373.
Geldoc XR Plus (Biorad) was used for detection.
ECL (relevant exposures)+ colorimetric overlays are shown.
The region that was used for the final figure has been highlighted in a dashed line square.

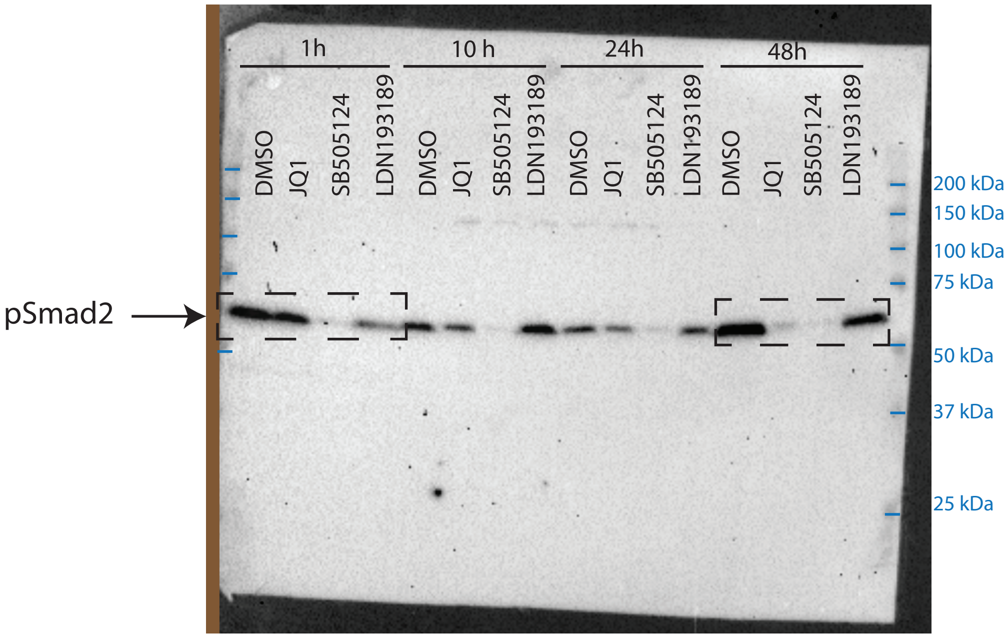

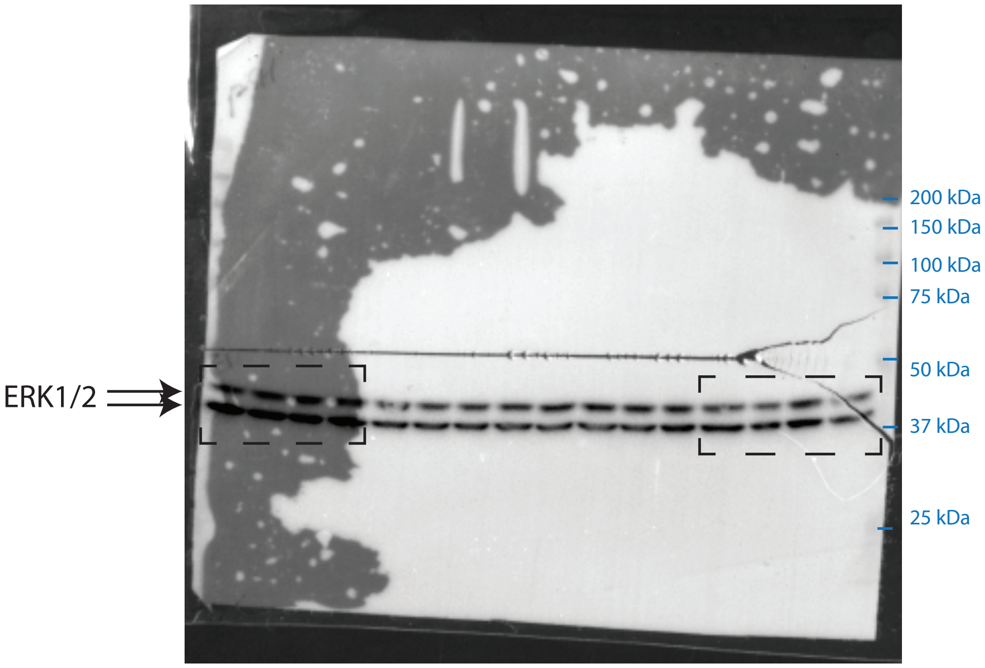

Supplement: Supplementary file 3 — Source Data for Expanded View [file EMBR-18-1108-s008.zip › EV2A_SD.pdf]
