# Supplementary material for: Brd4‐Brd2 isoform switching coordinates pluripotent exit and Smad2‐dependent lineage specification
Source: EMBO Rep. 2017 Jun 6;18(7):1108–22. doi: 10.15252/embr.201643534 (PMC5494510; doi:10.15252/embr.201643534)

Figure 1D

Gel: NuPAGE™ 4-12% Bis-Tris Protein Gels, 1.0 mm, 10-well.
Size marker: Precision Plus Biorad #161-0373.
Geldoc XR Plus (Biorad) was used for detection.
Overlays between a colorimetric image and a relevant ECL exposure are shown, unless stated otherwise.
The areas used for the final figure have been highlighted in a a dashed square.

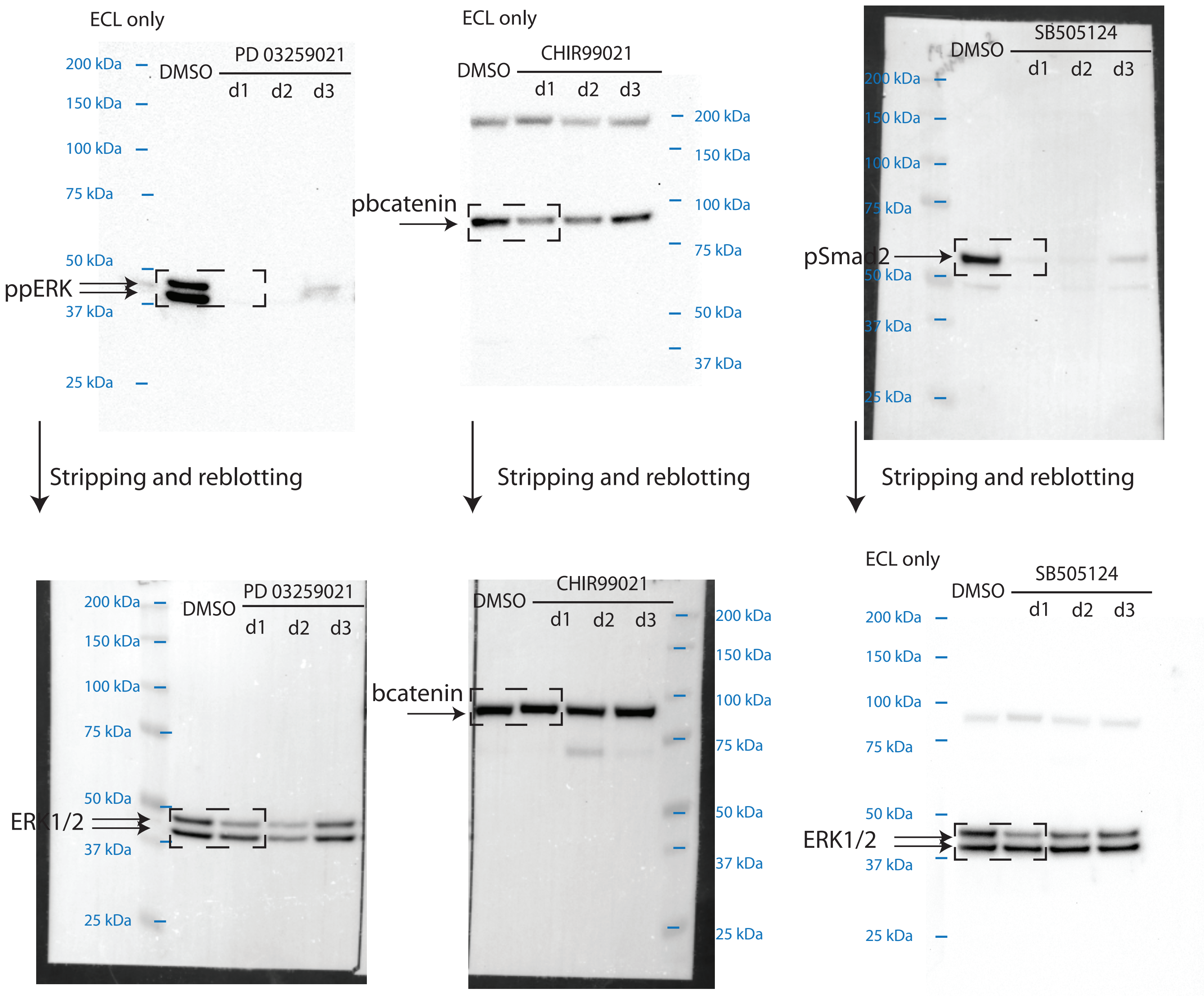

Supplement: Supplementary file 5 — Source Data for Figure 1 [file EMBR-18-1108-s003.pdf]
