# Supplementary material for: Brd4‐Brd2 isoform switching coordinates pluripotent exit and Smad2‐dependent lineage specification
Source: EMBO Rep. 2017 Jun 6;18(7):1108–22. doi: 10.15252/embr.201643534 (PMC5494510; doi:10.15252/embr.201643534)

Figure 2A

Gels: 10% polyacrylamide.
Size marker: Precision Plus Biorad #161-0373.
Membranes were cut at 50 kDa for blotting against different antibodies.
Geldoc XR Plus (Biorad) was used for detection.
ECL (relevant exposure) + colorimetric overlays are shown.
The region that was used for the final figure has been highlighted in a dashed line square.

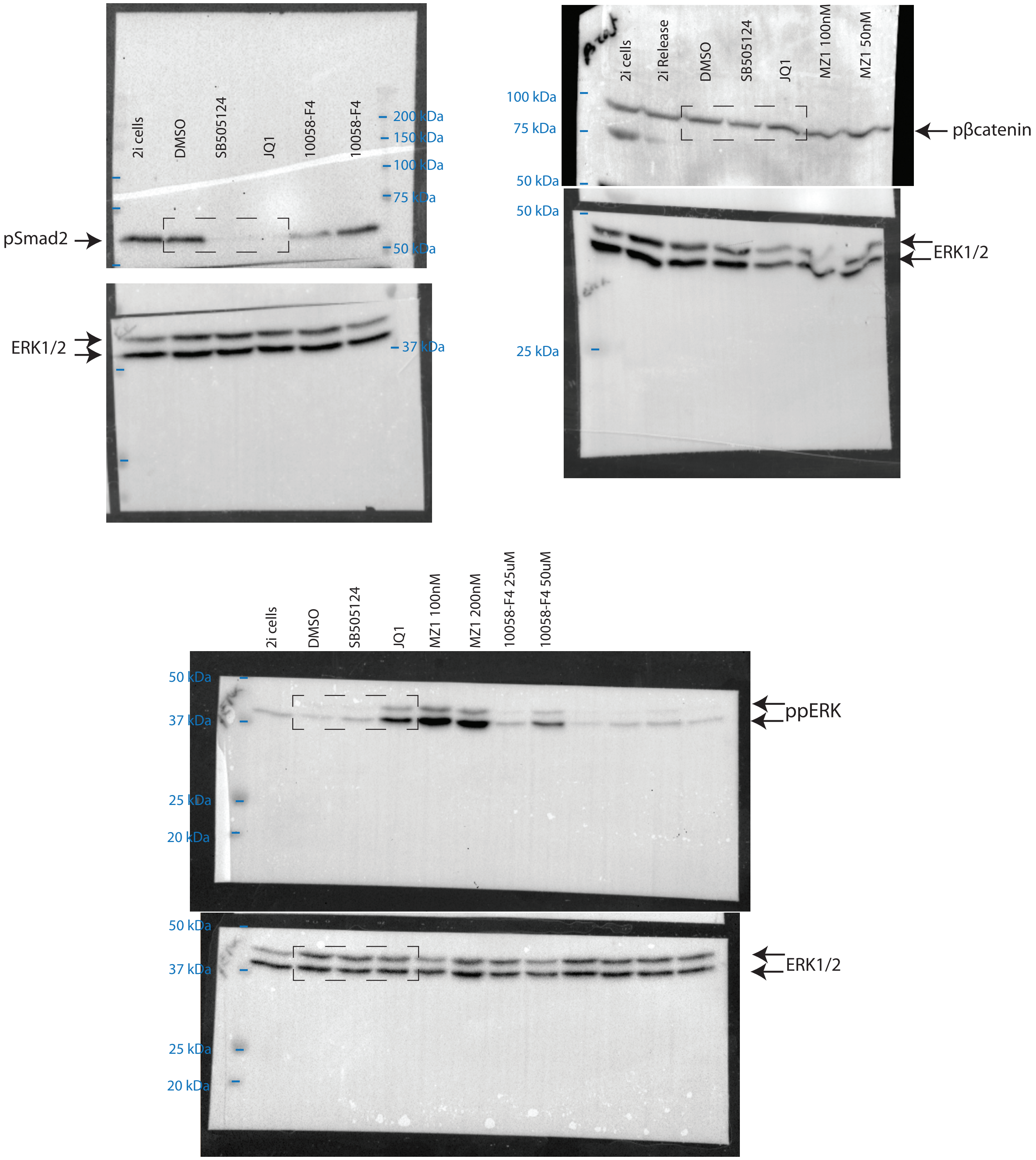

Supplement: Supplementary file 6 — Source Data for Figure 2 [file EMBR-18-1108-s004.zip › embr201643534-sup-0004-SDataFig2.pdf]
