# Supplementary material for: Brd4‐Brd2 isoform switching coordinates pluripotent exit and Smad2‐dependent lineage specification
Source: EMBO Rep. 2017 Jun 6;18(7):1108–22. doi: 10.15252/embr.201643534 (PMC5494510; doi:10.15252/embr.201643534)

Figure 6C

Gel: NuPAGE™ 3-8% Tris-Acetate Protein Gels, 1.0 mm, 12-well.
Size marker: Precision Plus Biorad #161-0373.
Geldoc XR Plus (Biorad) was used for detection.
ECL (relevant exposures)+ colorimetric overlays are shown.

Gel 1 (Brd4)

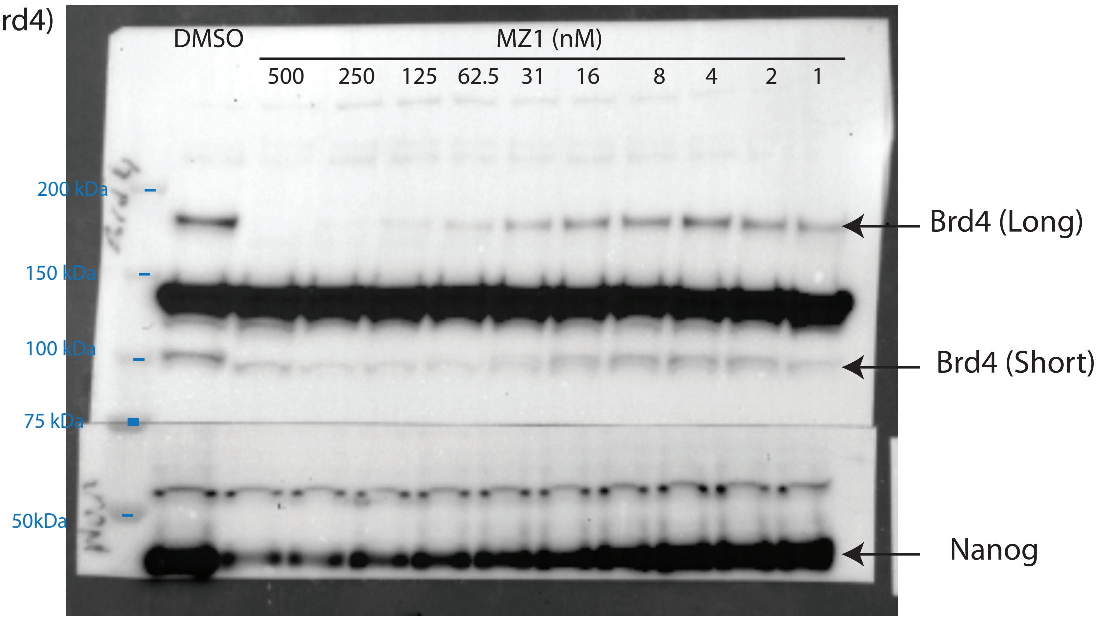

Gel 1 (Brd2)

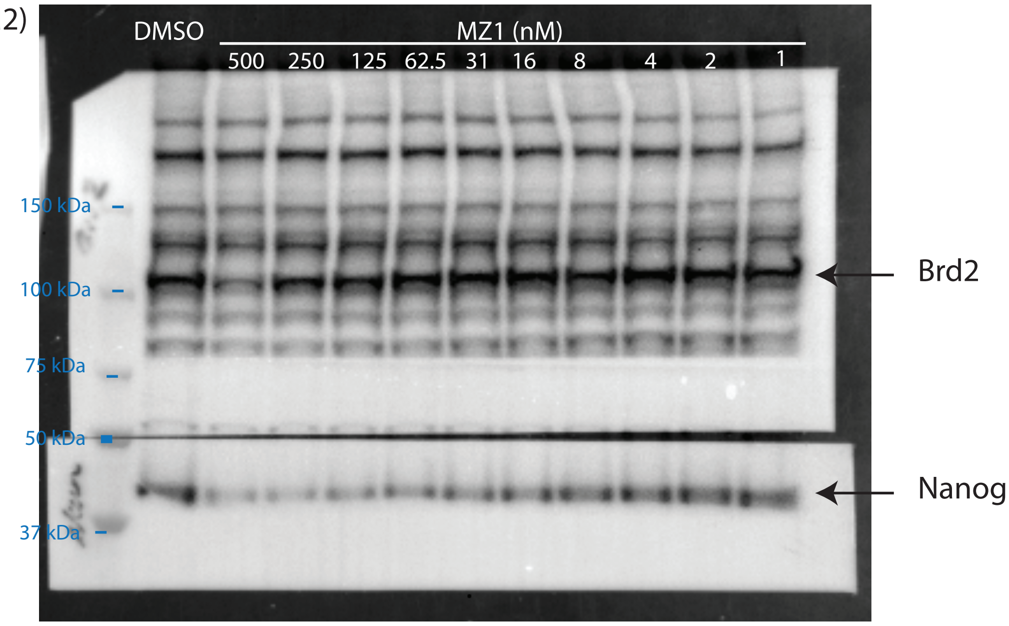

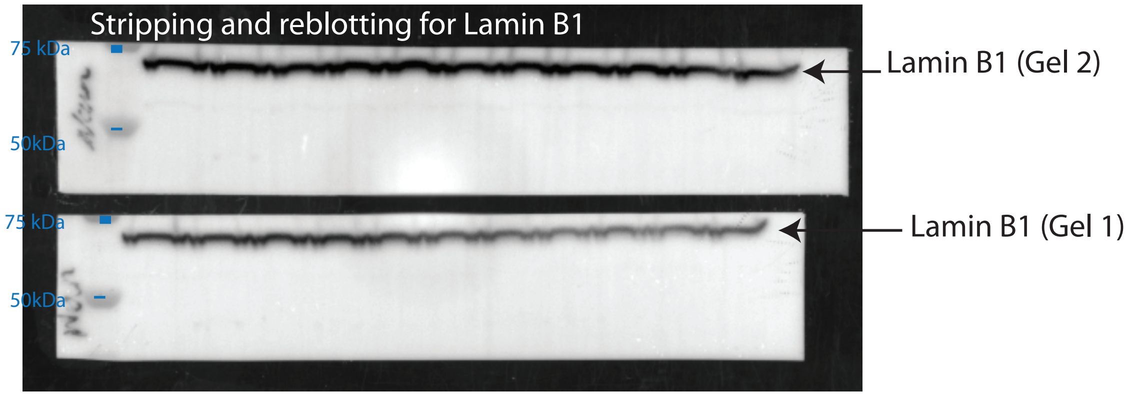

Supplement: Supplementary file 9 — Source Data for Figure 6 [file EMBR-18-1108-s007.zip › embr201643534-sup-0009-SDataFig6.pdf]
